# Comparison of Computational Models for Assessing Conservation of Gene Expression across Species

**DOI:** 10.1371/journal.pone.0013239

**Published:** 2010-10-08

**Authors:** Yupeng Wang, Kelly R. Robbins, Romdhane Rekaya

**Affiliations:** 1 Department of Animal and Dairy Science, University of Georgia, Athens, Georgia, United States of America; 2 Institute of Bioinformatics, University of Georgia, Athens, Georgia, United States of America; 3 Department of Statistics, University of Georgia, Athens, Georgia, United States of America; Institute of Infectious Disease and Molecular Medicine, South Africa

## Abstract

Assessing conservation/divergence of gene expression across species is important for the understanding of gene regulation evolution. Although advances in microarray technology have provided massive high-dimensional gene expression data, the analysis of such data is still challenging. To date, assessing cross-species conservation of gene expression using microarray data has been mainly based on comparison of expression patterns across corresponding tissues, or comparison of co-expression of a gene with a reference set of genes. Because direct and reliable high-throughput experimental data on conservation of gene expression are often unavailable, the assessment of these two computational models is very challenging and has not been reported yet. In this study, we compared one corresponding tissue based method and three co-expression based methods for assessing conservation of gene expression, in terms of their pair-wise agreements, using a frequently used human-mouse tissue expression dataset. We find that 1) the co-expression based methods are only moderately correlated with the corresponding tissue based methods, 2) the reliability of co-expression based methods is affected by the size of the reference ortholog set, and 3) the corresponding tissue based methods may lose some information for assessing conservation of gene expression. We suggest that the use of either of these two computational models to study the evolution of a gene's expression may be subject to great uncertainty, and the investigation of changes in both gene expression patterns over corresponding tissues and co-expression of the gene with other genes is necessary.

## Introduction

The biological functions of a gene, not only rely on its molecular composition and structure, but also on its spatiotemporal expression pattern. For example, duplicate genes, which are usually associated with highly consistent coding sequences but diverse biological functions, have only a weak correlation between rates of sequence and expression divergences [1]. Thus, it is of great importance to study both gene expression and sequence information to fully understand gene evolution.

Thanks to advances in microarray technology, the conservation/divergence of gene expression across species has been extensively and systematically assessed. However, results of such studies are often conflicting. Yanai et al. [2] concluded that no expression conservation exists in human and mouse orthologous gene pairs because the evolution in the expression profiles of orthologous gene pairs was shown to be comparable to that of randomly paired genes. In contrast, Liao and Zhang [3] found that the expression profile divergence for the majority of orthologous genes between humans and mice is significantly lower than expected under neutrality. Khaitovich et al. [4] suggested that the majority of expression divergences between species are selectively neutral and are non-functional adaptations, while Jordan et al. [5] suggested that gene expression divergence among mammalian species is subject to the effects of purifying selection and could also be substantially influenced by positive Darwinian selection. Yang et al. [6] found that broadly expressed genes have lower rates of gene expression profile evolution than narrowly expressed genes, while Liao and Zhang [7] proved the opposite. Furthermore, several studies found a strong correlation between gene expression divergence and coding sequence divergence [3], [8], [9], [10], [11], while other studies [2], [5], [12], [13], [14], [15] suggested little correlation between them.

Some of these conflicting conclusions on gene expression evolution may be due, in part, to improper comparisons of gene expression across genomes, such as direct comparisons of expression levels across probes or platforms, as suggested by Liao and Zhang [3]. Furthermore, cross-species microarrays hybridization may be problematic even when applied to closely related species [16], [17]. To overcome these limitations, indirect comparisons of gene expression across species have become a popular method for assessing conservation of gene expression. Liao and Zhang introduced the method of using relative mRNA abundance over 26 common tissues between humans and mice to make cross-species expression comparisons possible [3]. However, their method can be only implemented in closely related species, as it requires that the two microarray experiments sample orthologous tissues and use the same experimental procedures. Based on the conceptual framework of comparing co-expression patterns across species proposed by Ihmels et al. [18], Dutilh et al. [12], Tirosh and Barkai [13], and Essien et al. [19] used either all or part of the 1-1 orthologs as a reference set between species and computed the correlations of a gene's expression profile with those of the reference set for facilitating the study of assessing the degree of gene expression conversation across genomes. Theoretically, this framework can be applied to any species and any microarray data types. However, the use of the whole 1-1 ortholog set (WOS), as references in the study by Dutilh et al. [12], may be problematic because the subset of 1-1 orthologs with fast expression evolution may distort the true relationship of query genes. Tirosh and Barkai [13] identified this limitation and tried to minimize the influence of 1-1 orthologs with fast expression evolution by giving larger weights to orthologous pairs with conserved expression. Essien et al. [19] used the 1-1 orthologs in conserved co-expression networks (CCNs), instead of WOS, as a reference set between species.

The aforementioned methods represent two computational models for assessing conservation/divergence of gene expression across species: 1) comparison of gene expression patterns across corresponding tissues, and 2) comparison of co-expression of a gene with a reference set of genes. Although the separate application of either model has yielded significant biological insights [3], [7], [12], [13], [19], [20], [21], a systematic assessment of these models, especially their agreement with each other has yet to be reported. Until most recently, our group (Wang and Rekaya [22]) implemented both of these models to assess gene expression evolution between humans and mice. Surprisingly, we found little overlap between the conserved Gene Ontology (GO) terms detected by the two models. This observation has raised our concern about the usefulness and accuracy of the biological conclusions obtained using indirect comparison methods.

In this study, we assessed one corresponding tissue based method: Liao and Zhang's method [3] and three co-expression based methods: Dutilh et al.'s method [12], Tirosh and Barkai's method [13] and Essien et al.'s method [19], in terms of their pair-wise agreements. The comparisons were conducted using the human-mouse tissue gene expression data from Su et al. [23], one of the most frequently used dataset for the study of gene expression evolution.

## Methods

### Microarray data and annotations

A public human and mouse expression dataset was downloaded from GNF SymAtlas V1.2.4. at http://symatlas.gnf.org/SymAtlas/ (GEO accession number: GSE1133) [23]. The dataset consisted of 79 human and 61 mouse tissues using specially designed Affymetrix microarray chips (human: HG-U133A&GNF1H; mouse: GNF1M). The gene expression levels were obtained using MAS 5.0 algorithms [24]. To minimize the random effects of low expression values on estimating correlations [25], probes with an expression level <200 were removed from analyses. The annotation files for GNF1H and GNF1M were downloaded from GNF SymAtlas along with the data files. The annotation file for HG-U133A was downloaded from the Affymetrix website (http://www.affymetrix.com). To assign the Ensembl ID for each gene, the annotation files (humans: uniprot_sprot_human.dat; mice: uniprot_sprot_rodents.dat) were downloaded from the Uniprot FTP site at ftp://us.expasy.org/databases/uniprot/current_release/knowledgebase/taxonomic_divisions. The orthologous gene pairs between humans and mice were downloaded from the Ensembl FTP site (ftp://ftp.ensembl.org). Only 1-1 orthologs were considered in this study. The number of available 1-1 orthologous gene pairs was 7182, out of which 3142 had multiple probe sets. For a gene with multiple probe sets, the selection of a probe set that best represents the gene's expression profile according to a general rule has not been resolved yet [26]. Thus, in this study and in order to remove a potential additional source of variation in the data, the 1-1 orthologs with multiple probe sets were removed from analyses. The final number of human and mouse 1-1 orthologous gene pairs used for this study was 4040. These 4040 human-mouse1-1 orthologs constituted the WOS.

### Liao and Zhang's method for assessing conservation of gene expression between humans and mice

The expression data of 26 common tissues from two species were extracted and normalized by their relative abundance (RA) values calculated as:
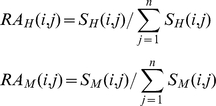
where *n* is the number of common tissues, *H* represents humans , *M* represents mice, and 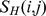 and 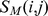 are the expression levels of gene *i* in human and mouse tissue *j*, respectively. The expression conservation (EC) for human-mouse orthologous pair *i* is calculated as:
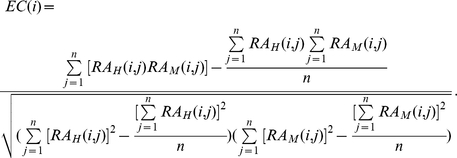
Its corresponding expression divergence measured by Euclidian distance is computed as:
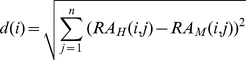



### Existing co-expression based methods for assessing conservation of gene expression

Expression datasets with different dimensions under different conditions between any two species, A and B, can be compared. The expression matrices, **A** and **B**, in species A and B respectively, are restricted to genes for which 1-1 orthology relationships have been identified and ordered accordingly (i.e., equivalent rows of the two matrices correspond to the expression profiles of a pair of orthologs):
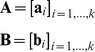
where 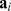 and 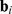 are the vectors of expression profiles for any pair *i* of 1-1 orthologs for species A and B, respectively, and *k* is the number of 1-1 orthologous gene pairs.

**A** and **B** are then converted into two pair-wise correlation matrices (PCMs), 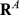 and 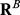, by computing the Pearson's correlation coefficient (denoted by PCC or *r*) between the expression profiles of each pair of genes over all conditions in each species separately:
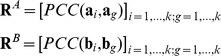


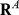
 and 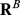 contain all the correlations between genes that have 1-1 orthology relationships. As they have the same dimension *k*, any row 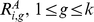 from 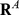 and any row 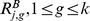 from 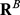 can be correlated. Dutilh et al. [12] defined the expression conservation (EC) for an orthologous gene pair *i* as:
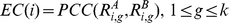
Tirosh and Barkai [13] suggested that a difference between 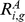 and 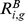 does not necessarily correspond to a difference in expression patterns of 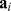 and 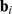, and thus when calculating the similarity between 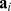 and 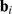, larger weight should be given to orthologous pairs whose expression has been conserved. To that aim, they developed the Iterative Comparison of Co-expression (ICC) algorithm. The ICC algorithm extends the above described procedure by iteratively refining the ECs using a weighted correlation, where the weight for each gene is given by the EC of that gene from the previous iteration:
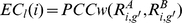
where
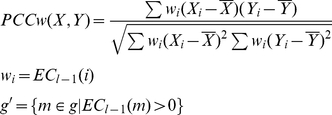
This iterative process is repeated until convergence:
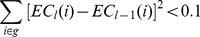
Essien et al. [19] computed the inter-species correlation, another expression of EC, in a similar way to how Dutilh et al. [12] computed the EC, except the reference ortholog set consisted of only the nodes in conserved co-expression networks (CCNs) between species. Thus, the EC by Essien et al.'s method can be computed as:
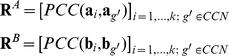


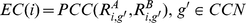
For co-expression based methods, the Euclidian distance between orthologs of gene *i* is computed as:
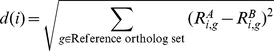



### Identification of reference ortholog set required for application of Essien et al.'s method

To apply Essien et al.'s method, the nodes of CCNs between humans and mice should be identified first. In this study, the identification of the nodes in CCNs, was performed via determination of conserved pair-wise co-expression between species, i.e. the expression profiles of a pair of genes are significantly correlated in both species. Intra-species background distributions of correlations were first constructed based on 20,000 random gene pairs. All two gene combinations were assessed for potential conserved co-expression. Gene pairs whose expression profiles were significantly correlated (*r* greater than a certain quantile *x* of the background correlation distribution, in both humans and mice) were selected as nodes of CCNs. Because the correlation cutoff value may affect the number of CNN nodes and in order to fully assess Essien et al.'s method, we varied the correlation coefficient threshold. Out of 4040 pairs of human-mouse 1-1 orthologs, 3390, 2424 and 1246 pairs were found as nodes of CCNs when the correlation threshold was set to 0.95, 0.975 and 0.99 quantile of the background distribution, respectively.

## Results

Because prior knowledge on the expression conservation for human-mouse orthologs is limited (expression conservation may not be associated with sequence conservation [5], [12], [13], [14], [15]), it is difficult to establish a benchmark for accurately evaluating the computational methods used for assessing expression conservation in terms of sensitivity and specificity. Given this difficulty and the purpose of this study, to examine whether different computational methods would generate consistent results on expression conservation, the performances of Liao and Zhang's method, Dutilh et al.'s method, ICC and Essien et al.'s method were evaluated based on their pair-wise agreements.

**Figure 1:**
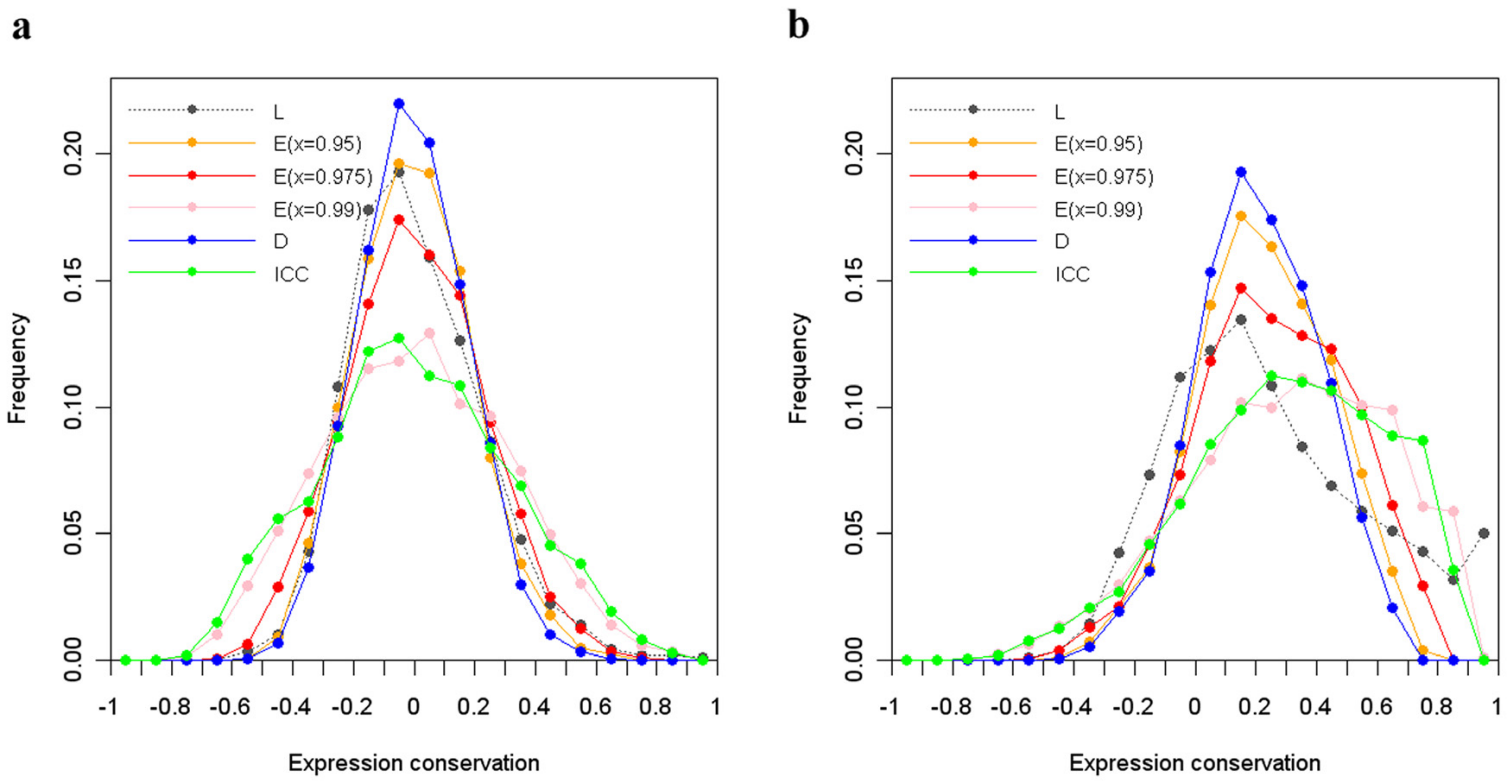
Comparison of the EC distributions for (a) human-mouse random gene pairs and (b) human-mouse 1-1 orthologs using Liao and Zhang's method (L), Dutilh et al.'s method (D), ICC and Essien et al.'s method (E).

Plots of the distributions of ECs for all human-mouse orthologous gene pairs and 4040 human-mouse random gene pairs, generated by different methods can be found in Figure 1. The means and standard deviations of these distributions are shown in Table 1. Generally, the comparisons of EC distributions between human-mouse orthologs and random gene pairs by different methods all prove the theory of non-random expression conservation of orthologs. This confirms that all the methods examined in this study are able to detect expression conservation. Note that there may be two steps in obtaining results of expression conservation of orthologs bioinformatically: the identification of orthologs and the measurement of expression conservation between orthologs. Liao and Zhang's method addresses issues related to the second step, while co-expression based methods can be applied to both steps. To demonstrate the usefulness of co-expression based methods in the first step, we re-generated the above results by disturbing the orthology relationships in the reference ortholog set (via permuting the order of columns of 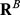). In this case, non-random expression conservation of orthologs is not observed (negative data are not shown), confirming that the 1-1 orthologs are a good reference gene set for co-expression based methods.

**Table 1:** Means and standard deviations of the EC distributions generated by different methods.

Feature of the EC distributions	Liao and Zhang's method	Co-expression based method				
		Dutilh et al.	ICC	Essien et al.		
				*x* = 0.95	*x* = 0.975	*x* = 0.99
Human-mouse random gene pairs						
Mean	0.004	−0.003	0.002	−0.001	0.004	0.007
Standard deviation	0.217	0.177	0.313	0.192	0.225	0.300
Human-mouse 1-1 orthologs						
Mean	0.253	0.209	0.305	0.226	0.258	0.312
Standard deviation	0.332	0.199	0.321	0.217	0.254	0.327

### Evaluation of the agreement between corresponding tissue based methods and co-expression based methods

Using Liao and Zhang's method as a reference, the three co-expression based methods generated variable EC distributions (Figure 1). For human-mouse random gene pairs, Essien et al.'s method at *x*(see the Methods section) = 0.975 generated an EC distribution that best approximated the EC distribution by Liao and Zhang's method; For the human-mouse orthologous gene pairs, when *x* = 0.975, Essien et al.'s method resulted in an EC distribution with a similar mean and a smaller standard deviation by comparison with Liao and Zhang's method. Within relation to Liao and Zhang's method, when *x* = 0.95 and *x* = 0.99, Essien et al.'s method tended to underestimate and overestimate the ECs respectively; Dutilh et al.'s method tended to underestimate the ECs and ICC tended to overestimate the ECs, though ICC had a comparable standard deviation to that obtained by Liao and Zhang's method. Additionally, the ECs of all human-mouse orthologous gene pairs generated by different co-expression based methods were correlated with those by Liao and Zhang's method. The correlation values are shown in Table 2. These results suggest that the co-expression based methods are only moderately correlated with the corresponding tissue based methods, and although Essien et al.'s method appears to best agree with Liao and Zhang's method, its performance is affected by the size of the reference ortholog set (i.e., number of the nodes in CCNs). Note that although co-expression based methods may generated different EC distributions, the ECs of human-mouse 1-1 orthologs computed by different co-expression based methods are highly correlated (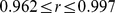).

**Table 2:** Correlations between Liao and Zhang's method and different co-expression based methods.

Correlation method	Dutilh et al.'s method	ICC	Essien et al.'s method		
			*x* = 0.95	*x* = 0.975	*x* = 0.99
Pearson's correlation	0.498	0.456	0.514	0.523	0.510
Spearman's correlation	0.477	0.440	0.492	0.502	0.498

The reliability of co-expression based methods for assessing cross-species conservation of gene expression may be greatly affected by the inclusion of fast evolving genes as references, as suggested by Tirosh and Barkai [13]. As such, a potential underlying problem with ICC is that, because 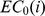 may be incorrectly computed using equal weights for all orthologous pairs which consist of both conserved and fast evolving genes (in expression), the weights given to the subsequent iterations may also be incorrect. Thus, an alternative approach to minimize the effects of fast evolving genes may rely on using a refined reference set which excludes fast evolving genes, such as Essien et al.'s method. The orthologs that are involved in CCNs have been shown to be more conserved in gene expression between species [27], which should be a better reference set for cross-species comparison of gene expression than WOS. Although it is reasonable to let the reference ortholog set consist of nodes in CCNs, the size of the reference set should be chosen appropriately because large reduction of dimensions may cause the correlation values to be unreliable while a too large size makes the performance of Essien et al.'s method approach that of Dutilh et al.'s method. Based on the analysis in this study, we would suggest that the size of the reference ortholog set range from 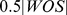 to 0.7 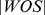.

### Problems in Liao and Zhang's method

Liao and Zhang's method was based on a subset of the microarray data, represented by the expression profiles over 26 human-mouse common tissues. However, the original human and mouse expression data cover 79 human tissues and 61 mouse tissues respectively. The potential problems for Liao and Zhang's method include 1) the similarity of gene expression profiles over only 26 common tissues may not reflect the expression conservation over all available tissues, and 2) common tissues are not the same tissues, i.e. tissues evolve between humans and mice.

Because there are no means of applying Liao and Zhang's method to the whole human and mouse tissue data, to quantify the effects of using the microarray data over only common tissues, we adopted an indirect approach: comparing co-expression based methods using the whole microarray data with the expression data over only common tissues (the same data used by Liao and Zhang's method), with the hypothesis that if the results on expression conservation do not differ significantly between the two types of expression data, the use of the expression data over common tissues should not be a factor affecting the assessment of expression conservation, which should be also true to Liao and Zhang's method. However, we found that the properties of EC distributions generated by co-expression based methods differ greatly between these two types of expression data (Table 3), and that the ECs of all human-mouse orthologous gene pairs inferred based on the whole microarray data and the expression data over 26 common tissues are only moderately correlated (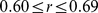), suggesting that the reduction from the whole microarray data to the expression data over 26 common tissues results in loss of information for assessing conservation of gene expression.

**Table 3:** Comparison of means of the EC distributions for human-mouse 1-1 orthologs based on the whole microarray data with the expression data over 26 common tissues by using co-expression based methods.

Co-expression based methods	Mean of the EC distribution		P-value by two-sample *t*-test
	Whole microarray data	Data over 26 common tissues	
Dutilh et al.'s method	0.209	0.168	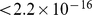
ICC	0.305	0.274	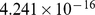
Essien et al.'s method (*x* = 0.975)	0.258	0.214	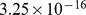

## Discussion

By applying co-expression-based methods to the expression data of 26 common tissues between humans and mice, i.e. the same data used by Liao and Zhang's method, a maximum agreement between corresponding tissue based methods and co-expression based methods can be estimated. Using this dataset, the ECs of all human-mouse 1-1 orthologs generated by different co-expression based methods were correlated with those generated by Liao and Zhang's method. Though these correlations were increased from (0.48–0.50) to (0.69–0.74), a maximum correlation of 0.74 is still far from a high agreement (say, *r*>0.9), suggesting that even if the same data are used, corresponding tissue based methods and co-expression based methods may still give different estimations of ECs.

In addition to expression conservation, expression divergence between species is also a measure for studying evolution of gene expression. Some studies used 1-EC as a measure of expression divergence [20], [21], and in this case the agreement between the assessed computational methods should be the same as the above analysis. Some studies used the Euclidean distance of expression profiles as a measure of expression divergence [5], [28], [29], [30]. We further reproduced the results by using Euclidean distances instead of ECs. However, negative correlations (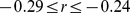) were observed between the Euclidean distances of human-mouse 1-1 orthologs computed by Liao and Zhang's method and those by co-expression based methods. This contradiction is not surprising as some previous studies have showed that Pearson's correlations and Euclidean distances may be completely uncorrelated [3], [5], [25]. To assess expression conservation, we would suggest the use of correlations instead of Euclidean distance because 1) they show agreements between different computational models; 2) unlike Euclidian distance, the scale of correlation ([−1, 1]) is not affected by different degrees of freedom. In addition to the potential contradiction between them, correlation and Euclidian distance have other limitations. They both measure the global similarity/divergence between gene expression profiles over multiple conditions/tissues, which may leave condition-specific / tissue-specific changes of gene expression undetected. However, some of these undetected changes may be caused by striking genetic evolution. Some studies [31], [32] have suggested that condition-specific / tissue-specific changes of gene expression should be also surveyed for fully understanding the mechanisms of gene regulation evolution.

In this study, we compared two popular computational models for assessing conservation of gene expression. The corresponding tissue based methods are only moderately correlated with co-expression based methods. All the assessed methods have limitations and thus, the use of a combination of Liao and Zhang's method and Essien et al.'s method (Essien et al.'s method appears better than Dutilh et al.'s method and ICC) is recommended. However, the two assessed computational models, which mainly capture the information on the global changes in gene expression patterns over orthologous tissues and in gene co-expression networks, reveal only part of the whole picture of gene expression evolution. Additionally, besides expression abundance as an indicator of gene expression behavior, expression breadth and specificity are also worth investigating [6], [7], [33]. Development of computational methods that properly model the divergence of expression breadth or specificity across species may be an important part of comprehensively assessing conservation of gene expression.
